# Evaluation of the Efficacy of the 755 nm Picosecond Laser in Eliminating Pigmented Skin Lesions after a Single Treatment Based on Photographic Analysis with Polarised Light

**DOI:** 10.3390/jcm13020304

**Published:** 2024-01-05

**Authors:** Piotr Zawodny, Nicole Wahidi, Paweł Zawodny, Ewa Duchnik, Elżbieta Stój, Wiola Rozalia Malec, Monika Kulaszyńska, Karolina Skonieczna-Żydecka, Jerzy Sieńko

**Affiliations:** 1 Medical Center Zawodny Clinic, Ku Słońcu 58, 71-047 Szczecin, Poland; estetic@estetic.pl (P.Z.); c.nicole@wp.pl (N.W.); ewa.duchnik@pum.edu.pl (E.D.); bubble.elisabeth@hotmail.com (E.S.); wiola.kuszlewicz@gmail.com (W.R.M.); 2 Department of Aesthetic Dermatology, Pomeranian Medical University in Szczecin, Powstancow Wlkp 72, 70-111 Szczecin, Poland; 3 Department of Biochemical Science, Pomeranian Medical University in Szczecin, Broniewskiego 24, 71-460 Szczecin, Poland; 4 Institute of Physical Culture Sciences, University of Szczecin, Piastow 40b, 71-065 Szczecin, Poland; jsien@poczta.onet.pl

**Keywords:** laser, skin, lesion

## Abstract

Introduction: Pigmentary changes can be bothersome and affect one’s well-being. Laser therapy has been shown to effectively treat such skin discolouration. We aimed to assess the utility of a 755 nm alexandrite laser in pigmented lesions removal. Materials and methods: A total of 109 patients aged 44.17 ± 8.2 years were enrolled and underwent laser treatment of facial skin hyperpigmentation. The efficacy was analysed on the basis of photographic diagnosis using the VISIA Complexion Analysis System. The following measures were assessed: (1) score; (2) feature count; (3) comparison figure. Results: A single laser treatment significantly improved the size and area of the lesion and decreased the number of lesion features. Parallelly, the overall skin condition significantly improved (*p* < 0.05). We found a statistical tendency of a higher feature count baseline, thus its change in men (*p* = 0.05 and 0.07, respectively), but failed to find any statistically significant associations (*p* > 0.05) between tested measures and skin phototypes and hyperpigmentation types. Age was also not correlated with the laser efficacy (*p* > 0.05). Conclusions: The use of the 755 nm laser is effective in reducing pigmented lesions.

## 1. Introduction

Today, we pay increasing attention to the appearance of our skin. However, pigmentary changes such as freckles or pigmentation spots can be extremely bothersome and affect our well-being. This is why a growing number of people are opting for treatment. Consequently, the removal of pigmented lesions such as sun spots, freckles, acne blemishes and other hyperpigmentation is becoming increasingly popular in aesthetic medicine [1]. Due to demand, the market for laser devices for this purpose is growing rapidly. Laser removal of pigmented lesions is a process that involves the precise and concentrated action of a laser beam within the skin to destroy the excessive accumulation of the pigment melanin. The main aim of the treatment is to evenly disperse or completely remove the pigment, resulting in an improved appearance and texture of the skin [1,2,3,4].

There are many different types of lasers and IPL (Intense Pulsed Light) devices used for the removal of pigmented lesions. We can divide lasers for this application into millisecond, nanosecond and picosecond technology. Hyperpigmentation treatments using millisecond technology include dye lasers, CO_2_ fractional lasers, Er:Glass, Er:YAG continuous wave and fractional lasers. Lasers working with nanosecond technology (the so-called Qswitch) include Nd:YAG lasers and alexandrite lasers, whilst the latest developments in picosecond technology are Nd:Yg and alexandrite lasers. Each of these types of lasers has its own unique characteristics that affect the efficacy, safety and recovery time of the treatment [3,4,5].

Ablative fractional lasers such as the CO_2_ laser and Er:YAG laser, and sub-ablative lasers such as the Tulowa laser or the Er:Glass non-ablative fractional laser, work by generating microchannels in the skin, allowing for intense skin remodelling and brightening of areas with discolouration. By precisely targeting selected sections of skin, fractional lasers minimise the risk of complications and reduce recovery time [3,6].

Picosecond lasers are characterized by a very short duration of individual pulses, which allows for the effective and safer removal of different types of pigmented lesions in a shorter period of time. Their action is based on breaking down the pigment particles into very small fragments, which are then removed by our body’s macrophages.

IPL (Intense Pulsed Light) technology is also used in the removal of pigmented lesions. Unlike lasers, which emit light at a single wavelength, IPL uses a broad spectrum of light, which allows for a less selective action on the skin, simultaneously removing different types of pigmented lesions and vascular lesions, and thermally stimulating collagen fibres [3]. In summary, there are many different types of lasers that can be used to remove pigmented lesions. The choice of the appropriate device depends on the individual needs of the patient and the assessment of the aesthetic practitioner.

Treating pigmented lesions with a picosecond laser, particularly with a wavelength of 755 nm (alexandrite laser), has become one of the most popular and effective ways to remove unwanted skin discolouration. This technology, due to its 755 nm wavelength and its affinity for melanin (selective photo thermolysis), is characterised by high precision and safety for the patient, making it an ideal solution for those wishing to remove their complexes [7,8,9,10].

In this article, we focus on evaluating the efficacy of picosecond laser therapy of pigmented lesions at 755 nm based on photographic diagnosis. An important part of this evaluation is the use of a VISIA device and a 365 nm filter to accurately diagnose pigmented lesions on the patient’s skin.

The aim of this study is to determine the effectiveness of the 755 nm picosecond laser in eliminating pigmented skin lesions after a single treatment based on photographic analysis with polarised light.

## 2. Materials and Methods

### 2.1. Patients

The effectiveness of the treatment of pigmented lesions with a 755 nm picosecond laser was evaluated in patients who underwent laser therapy at the Zawodny Clinic in Szczecin during the autumn-winter period from September 2018 to February 2022. A group of 109 patients aged 44.17 ± 8.2 years underwent laser treatment of facial skin hyperpigmentation. The group was dominated by women (n = 103, 94.5%). The majority of patients presented with hyperpigmentation due to mixed hyperpigmentation (n = 41; 37.6%; lesions included all: melasma, post-inflammatory and/or post-sun), which was followed by melasma (n = 38; 34.9%). Sun exposure (n = 17; 15.6%) and post-inflammatory hyperpigmentation (n = 13; 11.9%) were also reported. We determined the phototype according to the 6-degree Fitzpatrick scale based on history and skin reaction to sun exposure, and conducted a physical examination based on physical characteristics such as hair colour, skin complexion and eye colour, adopting generally used practical standards for such examinations. There were 74 (67.9%) patients with phototype II, followed by phototype I (n = 26; 23.9%) and III (n = 9; 8.3%).

### 2.2. Laser Treatment

A 755 nm wavelength alexandrite-type laser with picosecond technology was used to treat the patients in the study group. The laser system used is designed for the removal of tattoo-like pigmented lesions and hyperpigmentation. The 755 nm laser beam is strongly absorbed by melanin in the dermis, which results in the selective breakdown of pigmented lesions into small fragments without disturbing the continuity of the skin.

During the treatment, each patient wore protective goggles over their eyes; the number of pulses applied depended on the size of the facial skin area and the laser setting parameters varied according to skin phototype:

I–II phototype—energy density 2.08–2.83 J/cm^2^; light beam diameter 3.0–3.5 mm; pulse frequency 10 Hz; 4000–5000 pulses.

III phototype—energy density 2.08 J/cm^2^; light beam diameter 3.5 mm; pulse frequency 10 Hz; 4000–5000 pulses.

In all cases the duration of a pulse was 750 picoseconds.

The laser procedure was performed and the patients were informed about post-operative recommendations (moisturising the skin with Dexpanthenol cream for 3 days, avoiding direct sun/UV exposure and using sunscreens with SPF 50+ for 4 weeks) and possible complications (redness, swelling, temporary intensification of hyperpigmentation). The recovery process was also described to them: a possibility of slight swelling and redness of the skin on the first day, which should subside after 12–24 h, with the severity of the hyperpigmentation subsiding over a period of 3–7 days after treatment.

Importantly, the study patients underwent a series of several treatments which varied in number (3–8) and were carried out at different intervals (lack of patient subordination and consistency). Herein, the efficacy after one session is presented.

### 2.3. Laser Treatment Efficacy

To assess the efficacy of a single session with the 755 nm laser, the VISIA skin analysis system was used (Canfield Imaging Systems, Fairfield, NJ, USA). The first photograph was taken at baseline (before treatment). Three to four weeks after the procedure, patients who qualified for the study were asked to return to the Clinic for a follow-up visit to analyse the effect of the procedure and to take photographs (endpoint).

**Figure 1:**
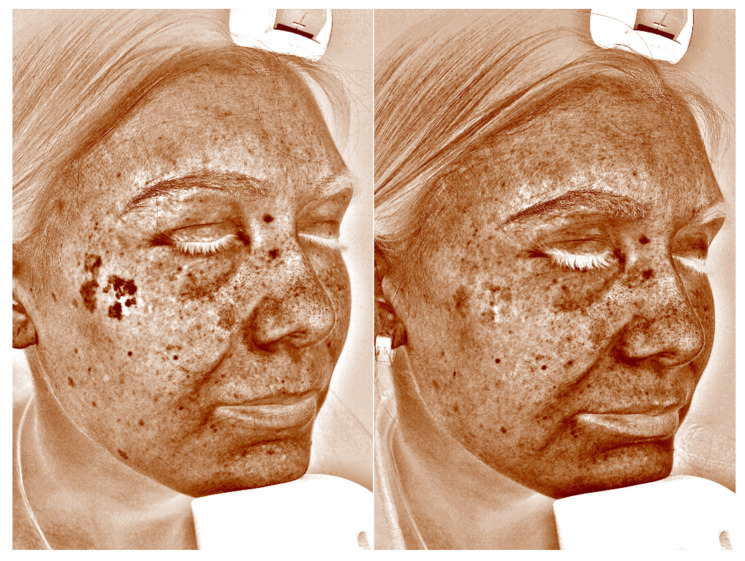
VISIA comparison photograph: exemplary effect of a single picosecond laser treatment with a wavelength of 755 nm in polarized light.

The VISIA skin analysis computer system determines the condition of the skin objectively and reproducibly using special numerical algorithms. The photographic device consists of a high-resolution camera and polarized light placed in a special capsule to ensure optimal and reproducible lighting conditions. Data are sent to a connected computer with special VISIA software (version 6.3.3). This system uses RBX technology (red/brown sub-surface analysis), which determines the number of pigmented lesions exceeding an established standard (brown areas) and enables an analysis of the status of these lesions (score). The software then performs a comparative analysis of the patient in question with a group of people in the database of the same age and skin phototype. Exemplary photographs are depicted in Figure 1. More can be found in the Supplementary Material presenting before and after natural light photographs with corresponding VISIA images.

The patient’s preparation for photography was as follows: double make-up removal and removal of impurities from the facial skin with a mild micellar make-up remover, and toning of the facial skin with a non-alcoholic toner. The cosmetics for the make-up removal were chosen in such a way that they would not irritate the skin before the photography. Facial skin cleansing was performed with a physiological pH gel, the main composition of which was thermal water. The face was then washed with a micellar lotion for sensitive skin with a physiological pH. The steps were repeated twice. Ten minutes after cleansing the face, photographs were taken using a skin analysis system. The same pattern of patient preparation for photography allowed for reproducibility and reliability of the results. Patient photographs were taken from the right, left, and of the face profile. However, the side with the most pigmented lesions was analysed numerically.

### 2.4. Outcome Measures

The following outcomes generated in the VISIA system were used to evaluate the laser efficacy:

1. Score—absolute result providing a comprehensive measure of the impact the feature has on the patient’s complexion. These results include the total size and area, as well as the intensity of the detected elements of the analysed feature.
2. Feature count—the number of elements of a trait being assessed, regardless of the size and intensity of each instance.
3. Comparison figure—a percentage result of the patient’s complexion analysis, showing a comparison of individual values for people with similar features (useful in providing a baseline assessment of the patient’s overall complexion).

For each of the abovementioned parameters a delta was calculated (endpoint value minus baseline value).

We also assessed the frequency of adverse exceeding intensity and duration of the previously indicated temporary symptoms in the form of slight redness, swelling 24 h after the treatment and a temporary (3–7 days) increase in pigmentation.

### 2.5. Statistical Analyses

To assess the distribution of continuous variables, the Shapiro–Wilk normality test was adopted. Descriptive statistics were then reported using median and interquartile ranges (IQR). Qualitative variables were presented as numbers and percentages. To test laser efficacy, a paired sample Wilcoxon test was employed. In order to find the association between the efficacy and skin phototype, a Kruskal–Wallis test was used. A correlation between outcomes and participants’ age was calculated by means of the Spearman method. The significance level for type I error was set at 0.05, and the calculations were performed using MedCalc statistical software version 22.006 (Ostend, Belgium).

## 3. Results

The primary outcome of our study was to assess whether a single session using a 755 nm picosecond laser is efficient in eliminating pigmented skin lesions. In order to evaluate this, we compared the values of each outcome endpoint vs. baseline in a paired manner. We were able to show that a single laser treatment significantly improved the size and area of the lesion and decreased the number of lesion features. Parallelly, the overall skin condition significantly improved. The results are placed in Table 1.

**Figure 2:**
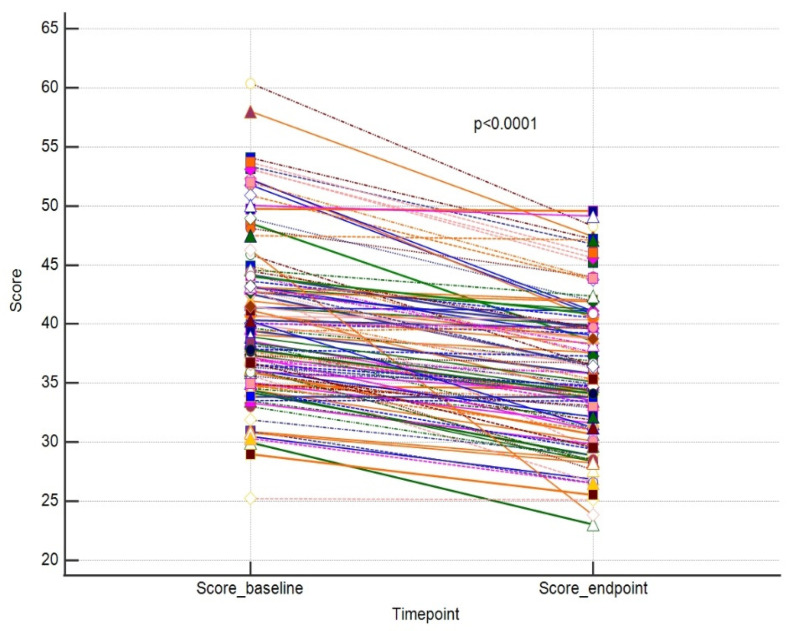
A paired comparison regarding VISIA score. Different line colours represent individual’s data.

**Figure 3:**
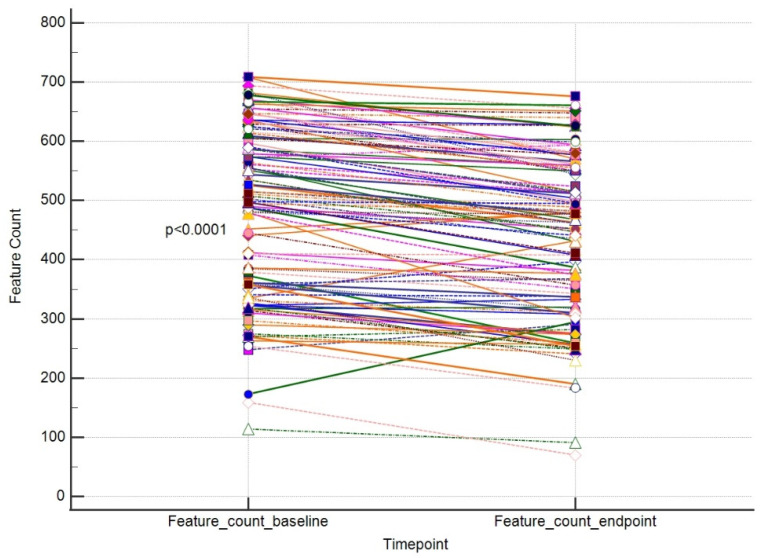
A paired comparison regarding VISIA Feature Count. Different line colours represent individual’s data.

**Figure 4:**
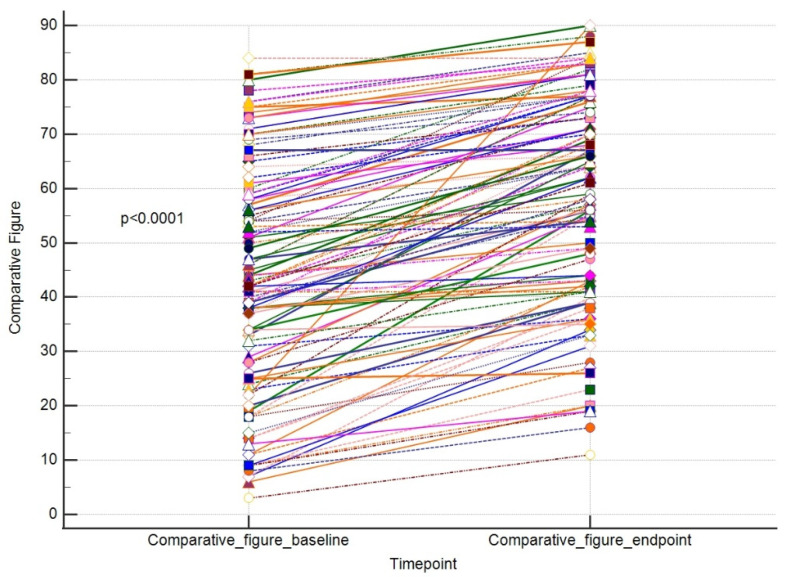
A paired comparison regarding VISIA comparative figure. Different line colours represent individual’s data.

The results for these outcomes are presented as dot-line diagrams in Figure 2, Figure 3 and Figure 4.

Next, we were curious to identify a variable that might be responsible for the effect size of a laser treatment. First, we tested if gender significantly influenced outcome measures (Table 2). We found a statistical tendency of a higher feature count baseline, thus its change in men (*p* = 0.06 and 0.08, respectively). As we collected data on the skin phototypes of all recruited patients, we looked for the association between this parameter and a difference in the tested parameters. We observed that a median feature count change was highest in the case of phototype I, and the highest improvement in VISIA score was for phototype III, so as for the overall change in a skin condition. However, we failed to find any statistically significant associations, as shown in Table 3.

**Figure 5:**
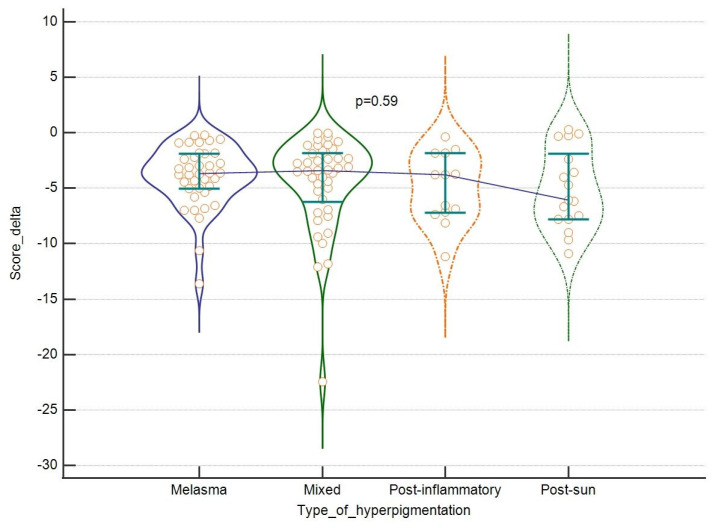
The efficacy of the laser: score change, (in regard to the type of hyperpigmentation. Violin plots depict medians and IQRs. Medians are connected with horizontal lines. Orange dots present individual cases.

**Figure 6:**
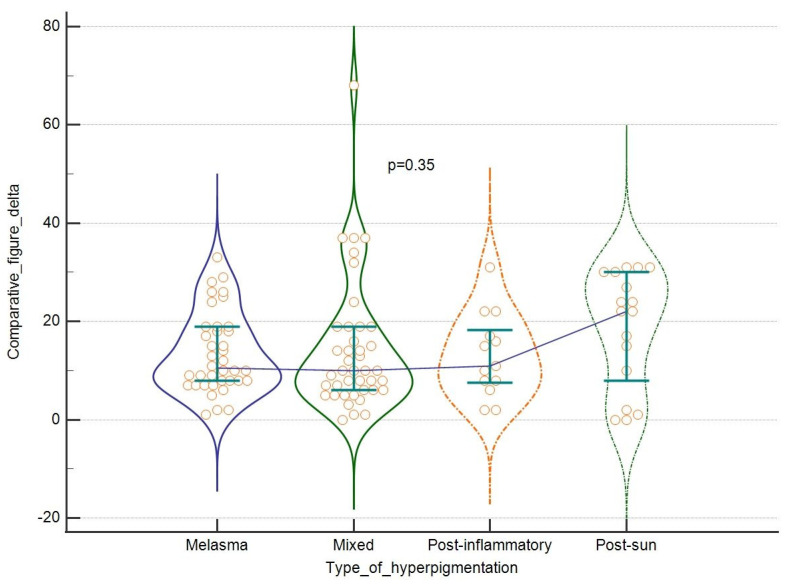
The efficacy of the laser: comparative figure change in regard to the type of hyperpigmentation. Violin plots depict medians and IQRs. Medians are connected with horizontal lines. Orange dots present individual cases.

Also, taking into consideration the type of hyperpigmentation, we were able to see some more intensive improvements in the case of score change for melasma and mixed hyperpigmentation, feature count change for melasma and post-inflammatory lesions, and comparative figure for post-sun hyperpigmentation. These differences were however not statistically significant, as shown in Figure 5 and Figure 6. Finally, no correlation was found between the tested parameters at baseline and the outcomes and patients’ age. For results, see Table 4.

No adverse events were reported by study participants.

## 4. Discussion

Hyperpigmentations are skin problems that are commonly diagnosed and treated in medical practices. According to our observations, the most common pigmentation disorders are post-inflammatory hyperpigmentation, freckles and melasma. These are generally benign lesions but can sometimes be distressing for patients. An adequate yet thorough dermatological history and skin examination are helpful in ruling out malignant tumours and their sequelae. Amongst a plethora of aesthetic cosmetology options to improve skin look, lasers are widely used. The removal of pigmented lesions might for instance involve lasers with pulse durations in milliseconds at 532 nm, 1064 nm, 10,600 nm, 2940 nm and 1550 nm, and IPL devices in the 560–1200 nm range. Of note, picosecond lasers with wavelengths of 755 nm and 1064 nm have been used for this purpose. The mechanism of removing pigmented lesions with lasers whose pulse lasts milliseconds involves thermal destruction (burning of the pigment), while picosecond lasers act photomechanically by breaking down the pigment of small particles with minimal thermal effect. The latter reduces the formation of inflammation and the risk of post-inflammatory hyperpigmentation, making this instrument an attractive option for health professionals of aesthetic cosmetology and dermatology.

To the best of our knowledge and own medical practice, one of the most effective lasers for removing pigmented lesions is a device with a wavelength of 755 nm and a single pulse duration in the picosecond range. For practitioners of aesthetic medicine, the criterion for the choice of laser technology is the effectiveness and safety of the procedure.

Of course, most pigmentation removal procedures are carried out in series and with additional complementary treatment, but in this case the effectiveness is influenced by many additional factors, such as: long-term external complementary treatment, medication taken, post-treatment care, UV exposure in the following months, other anti-ageing therapies, etc. Therefore, in our opinion, it is valuable for the practitioner to evaluate the technology, the effectiveness of which can be assessed after individual application.

In the present study, we attempted to analyse if a single 755 nm picosecond laser session in patients with skin colour defects is effective. Here, we provide evidence on the effectiveness of the treatment but without any dependency of gender, patient’s age and skin type after only one session. The results help to dispel any doubts about this innovative method of removing unwanted skin discolouration.

In our study, we utilized an objective method to evaluate the skin condition—the VISIA Skin Analysis System—to help visualise skin features that may not be visible to the human eye [11]. This has been used successfully by other researchers. For instance, in their study, Goldsberry et al. evaluated the VISIA System itself as a tool to help patients better understand their skin complaints [12]. Henseler, in a study on the accuracy of the VISIA System, also came to similar conclusions and found the VISIA device provides satisfactory results in assessing patients’ skin conditions [11]. With the help of this device, we evaluated three major outcomes, i.e., score—depicting the total size and area of a lesion; feature count—the number of elements of a trait; and finally, a comparison figure—a percentage result characterizing a patient’s overall skin condition. After a single laser session, all of these parameters were significantly improved (*p* < 0.05), but the efficacy was not dependent on the patients’ gender, skin phototype and their age.

The efficacy of the picosecond laser has been reported in the scientific literature. Wang et al. used the VISIA approach to analyse the results of their study on the removal of hyperpigmentation using a picosecond laser and creams containing fluocynolone acetonide, hydroquinone and tretinoin. They demonstrated that alexandrite picosecond laser treatment showed similar efficacy in the treatment of melasma as the use of the aforementioned creams [13]. Chan et al. investigated the efficacy of a 755 nm picosecond laser for the treatment of benign pigmented lesions in Asians; Q-switched lasers often put this group at risk of post-inflammatory hyperpigmentation. Thirteen patients entered the study and the number of treatments ranged from 1 to 7. The lesions studied were nevus of Ota, cafe-au-lait spots, Becker nevus, lentigines and moles. In the patient with nevus of Ota, complete regression of the lesion was achieved after four treatments, while another two patients had excellent results after three and four treatments, respectively. In patients with cafe-au-lait spots, an average to good effect was achieved after one to seven treatments. In none of the patients was post-inflammatory hyperpigmentation observed after treatment. In summary, the 755 nm picosecond laser has high efficacy and a low risk of post-treatment complications [7].

An exceptional efficacy of hyperpigmentation removal after the first treatment with 755 nm occurs as per our subjective observations of our group of professionals. Thus, in order to standardise the study group and to objectivise the efficacy of such treatment, we decided to analyse the impact after only one session. Nevertheless, we herein report that the study patients underwent a series treatments which varied in number (3–8) and were carried out at different intervals (lack of patient subordination and consistency). Nevertheless, the number of treatments needed to satisfy the patient and the laser practitioner depends on a number of factors, such as the amount and depth of melanin. Of note, Yu et al. compared the efficacy of treatment of acquired bilateral nevus of Ota with an alexandrite picosecond laser and an alexandrite Q-switched laser. The study group consisted of 30 patients. Each received three treatments at six-month intervals. One side of the face was treated with the picosecond laser and the other with the Q-switched laser. The efficacy of both lasers was determined by patient self-assessment and visual assessment at the in-office consultation. The area treated with the picosecond laser achieved significantly better clearance (3.73 vs. 2.4) while experiencing less pain (4.47 to 5.16). The incidence of post-inflammatory hyperpigmentation reached 27.77% and 54.44% for picosecond and Q-switched laser treatment, respectively, and the duration of post-inflammatory hyperpigmentation was 1.32 and 1.74 months, respectively. This illustrates the significantly better clinical outcome of the picosecond laser and fewer side effects than the Q-switched laser [14]. Similar issues were studied by Ge et al. When comparing the performance of the two lasers, better clinical results as well as fewer side effects were indicated with the picosecond laser with a wavelength of 755 nm [10]. Another thing to consider regarding the comparisons with other studies with similar aims is other descent, thus different phototypes distribution [7,15,16,17] across studied population. Thus, overall efficacy needs to be taken with caution. A meta-analytic approach regarding this, with subgroup analyses by skin phototype, would be needed to clearly define the effect size.

A very important issue is the occurrence of side effects after laser treatments. The analysis of the studies conducted by the above-mentioned authors shows that treatments with the 755 nm wavelength alexandrite laser significantly reduce the risk of side effects and adverse reactions compared to Q-switched nanosecond lasers. Sakio et al. also studied the efficacy and safety of the picosecond laser in the treatment of Ota nevi. The study group consisted of 15 patients, 12 women and three men. Outcome was assessed by clinical photography taken before treatment and three months after the last treatment. All subjects showed an improvement in nevus reduction, with 10 subjects having an excellent outcome and five having a good outcome. The interval between treatments averaged 4.1 months and the average total treatment period was 11.6 months. Acute complications such as swelling, pain and scab formation were mild and all completely resolved within 14 days. No complications in the form of scarring, texture change or hypopigmentation were reported. The study reported a small percentage of post-inflammatory hyperpigmentation, which resolved within three months in 78.9% of all subjects. Sakio et al. believe that the treatment of Ota nevi with a picosecond laser achieves better results, with a shorter treatment interval and shorter treatment time, and with fewer complications. More importantly, treatment with this laser involves less damage to the epidermis and the possibility of breaking down larger pigment particles with an ultra-short pulse than with a Q-switched laser. As a result, patients’ quality of life may be improved [8]. Interesting conclusions were also reached by Yang et al. in a study of 20 Chinese women who had freckles on their faces. One side of the face was treated with a Q-switched alexandrite laser (control group) and the other with a picosecond laser (experimental group). The level and duration of adverse effects such as erythema, swelling, discolouration and blistering were studied; however, these were not statistically significantly different between the two groups. Almost all subjects observed mild to moderate swelling after treatment, which resolved after one to two days. Although similar treatment effects were observed with the aforementioned lasers in the above study, it can be speculated that picosecond laser therapy causes less photothermal damage [18].

Alegre-Sanchez et al. unanimously report that patients enrolled in the study are satisfied with the treatment results and the picosecond alexandrite laser is effective in treating both flat pigmented lesions and those raised above the skin surface [19].

Another type of hyperpigmentation is hyperpigmentation in the infraorbital region, the treatment of which with the alexandrite picosecond laser was addressed by Vanaman Wilson et al. A study group of ten respondents achieved significant improvement in the appearance and lightening of hyperpigmentation after a series of three treatments [9]. According to a study by Weiss et al., the alexandrite picosecond laser with a wavelength of 755 nm performs very well in cases of argyria. This is a blue-grey discolouration caused by chronic exposure to silver. Although the condition is asymptomatic, it often looks disfiguring and causes psychological discomfort. A woman who had taken an oral colloidal silver solution for a period of five years was examined. Blue-grey patches were localised in the face, neck, back, limbs and oral mucosa. The patient achieved complete resolution of the argyria after laser treatment [20]. In contrast, Jakus et al. studied the treatment of hyperpigmentation caused by long-term minocycline therapy. Two patients were treated with a picosecond laser on one part of the face and a Q-switched neodymium-yag laser on the other part. The third respondent was treated only with the picosecond laser. The results showed greater efficacy with the picosecond laser [21]. Similar conclusions were reached by Sasaki et al. who also looked at the efficacy of minocycline therapy-induced pigmented lesions. The studied woman was treated for pigmented lesions on both lower limbs, which resolved after one treatment with a picosecond laser with a pulse duration of 750 picoseconds [22]. Coincidentally, Barrett et al. also used a picosecond laser to treat pigmented lesions resulting from long-term minocycline therapy, which contributed to the resolution of pigmentation [23]. Based on the above results, it can be concluded that the use of the picosecond laser with a wavelength of 755 nm is safe for patients, the number of treatments needed to obtain satisfactory results is much lower than with other lasers, and side effects are non-existent or mild and disappear after a few days without a recovery period.

Our study has strengths and limitations. First, in many studies evaluating 755 nm laser efficacy, the authors used subjective efficacy assessments, such as the grading system—quartile improvement scale [14,24], visual clinical improvement score [25], a visual analogue scale consisting of six levels of treatment response according to percentage of pigmentary lightening [26,27], and self-reports [28]. Our study utilised the objective VISIA system to evaluate the efficacy of the laser procedure, with three different outcome measures: score, feature count and comparative figure. Additionally, a number of persons recruited for our study exceeds what has been described so far in the literature. For instance, Woraphong Manuskiatti [29] randomized 18 patients, Ng et al. recruited 20 patients [30] and Bonan et al. enrolled 63 patients [24]. Chesnut et al. provided data on three patients [31] so as Huang [32], whilst Fabi et al. evaluated the efficacy in 20 patients [33] and Yiming Li analysed 37 persons [34]. A case study was published by Iwayama et al. [35], Kok and Chua [36] and Rivers et al. [37]. Alster and Gupta published a case series of six persons [38], whilst Pindado-Ortega et al. evaluated the laser efficacy in two persons [39]. To the best of our knowledge the highest number of patients was analysed by Levin et al. [26] (n = 70) and Zhang et al. [28] (n = 86). In conclusion, we provide the first report on 755 nm laser efficacy in 109 patients. Finally, we provide data on the efficacy of the 755 nm laser regarding gender, type of lesion, skin phototype and age.

Nevertheless, we confirm that an imbalance in gender distribution and low number of Fitzpatrick grade 3 representatives are the major limitations of our study.

## 5. Conclusions

According to our research, the 755 nm alexandrite laser with picosecond technology has been shown to be effective in the treatment of pigmented facial skin lesions. The results obtained using the VISIA skin analysis system show a significant improvement in skin quality in all patients, but the efficacy is not dependent on age, gender, type of hyperpigmentation or phototype. The use of the 755 nm wavelength proved effective in reducing pigmented lesions, as a significant reduction in the number of brown areas and the severity of melanin saturation of hyperpigmentation was observed in the entire study group.

## Figures and Tables

**Table 1:** The efficacy of a single picosecond laser treatment.

VISIA Variable	n	Median Baseline	Median Endpoint	Median Paired Difference	*p*-Value
Score	109	38.926	35.302	−3.752	<0.0001
Feature count		506	463	−40	<0.0001
Comparative figure		42	61	11	<0.0001

**Table 2:** The outcomes by gender.

Variable	Females (n = 103)		Males (n = 6)		*p*-Value
	Median	IQR	Median	IQR	
Score_baseline	38.56	35.63–43.21	42.09	37.83–47.49	0.24
Score_endpoint	35.12	31.04–39.70	38.63	34.73–41.94	0.14
Score_delta	−3.77	−6.78–−1.96	−2.79	−6.54–−0.39	0.41
Feature_count_baseline	503.00	343.50–595.50	631.00	487.00–678.00	0.06
Feature_count_endpoint	463.00	312.50–554.50	547.00	441.00–555.00	0.22
Feature_count_delta	−38.00	−71.00–15.25	−58.00	−98.00–52.00	0.08
Comparative_figure_baseline	42.00	26.50–59.00	45.00	39.00–53.00	0.99
Comparative_figure_endpoint	61.00	42.00–76.75	60.00	43.00–70.00	0.65
Comparative_figure_delta	11.00	7.00–21.25	12.50	2.00–18.00	0.67

**Table 3:** The VISIA measures (deltas) by skin phototype.

Factor	n	Minimum	25th Percentile	Median	75th Percentile	Maximum	*p*-Value
Score delta							
I	26	−10.6	−6.07	−3.805	−0.9	−0.02	0.16
II	74	−22.43	−6.57	−3.535	−1.85	0.31	
III	9	−12.1	−11.14	−5.81	−3.855	−1.59	
Feature Count_delta							
I	26	−138	−99	−61.5	−26	97	0.11
II	74	−178	−70	−37	−16	122	
III	9	−94	−76.25	−34	−2.75	39	
Comparative Figure_delta							
I	26	0	8	15	19	37	0.47
II	74	0	7	10	19	68	
III	9	5	7.5	19	26.25	32	

**Table 4:** The correlation between outcomes and age of participants.

	Age	
Score_baseline	Correlation coefficient	0.124
	*p*	0.1994
	n	109
Score_endpoint	Correlation coefficient	0.155
	*p*	0.1079
	n	109
Score_delta	Correlation coefficient	0.092
	*p*	0.3415
	n	109
Feature_count_baseline	Correlation coefficient	0.133
	*p*	0.1689
	n	109
Feature_count_endpoint	Correlation coefficient	0.092
	*p*	0.3418
	n	109
Feature_count_delta	Correlation coefficient	−0.052
	*p*	0.5939
	n	109
Comparative_figure_baseline	Correlation coefficient	−0.061
	*p*	0.5305
	n	109
Comparative_figure_endpoint	Correlation coefficient	−0.053
	*p*	0.586
	n	109
Comparative_figure_delta	Correlation coefficient	−0.02
	*p*	0.8329
	n	109

## Data Availability

The data presented in this study are available on request from the corresponding author.

## Supplementary Materials

The following supporting information can be downloaded at: https://www.mdpi.com/article/10.3390/jcm13020304/s1, Figure S1: The efficacy of single 755 nm picosecond laser session. Pictures present en face profile before and after single teratment in visible and polarised light respectively.
