# Supplementary material for: Evaluation of the Efficacy of the 755 nm Picosecond Laser in Eliminating Pigmented Skin Lesions after a Single Treatment Based on Photographic Analysis with Polarised Light
Source: J Clin Med. 2024 Jan 5;13(2):304. doi: 10.3390/jcm13020304 (PMC10816936; doi:10.3390/jcm13020304)
Supplement: Supplementary file 1 [file jcm-13-00304-s001.zip › jcm-2736242-supplementary.pdf]

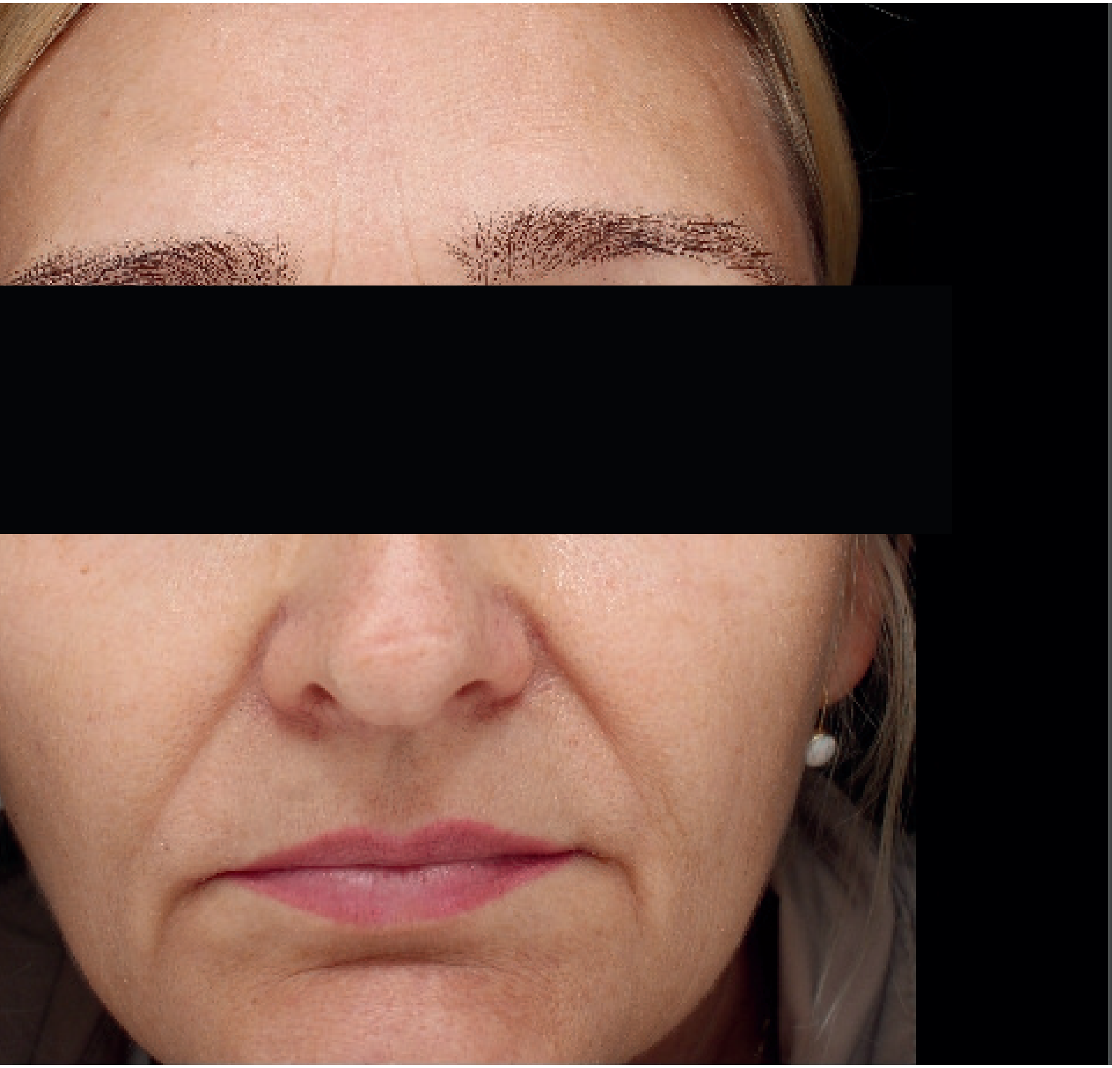

BEFORE TREATMENT

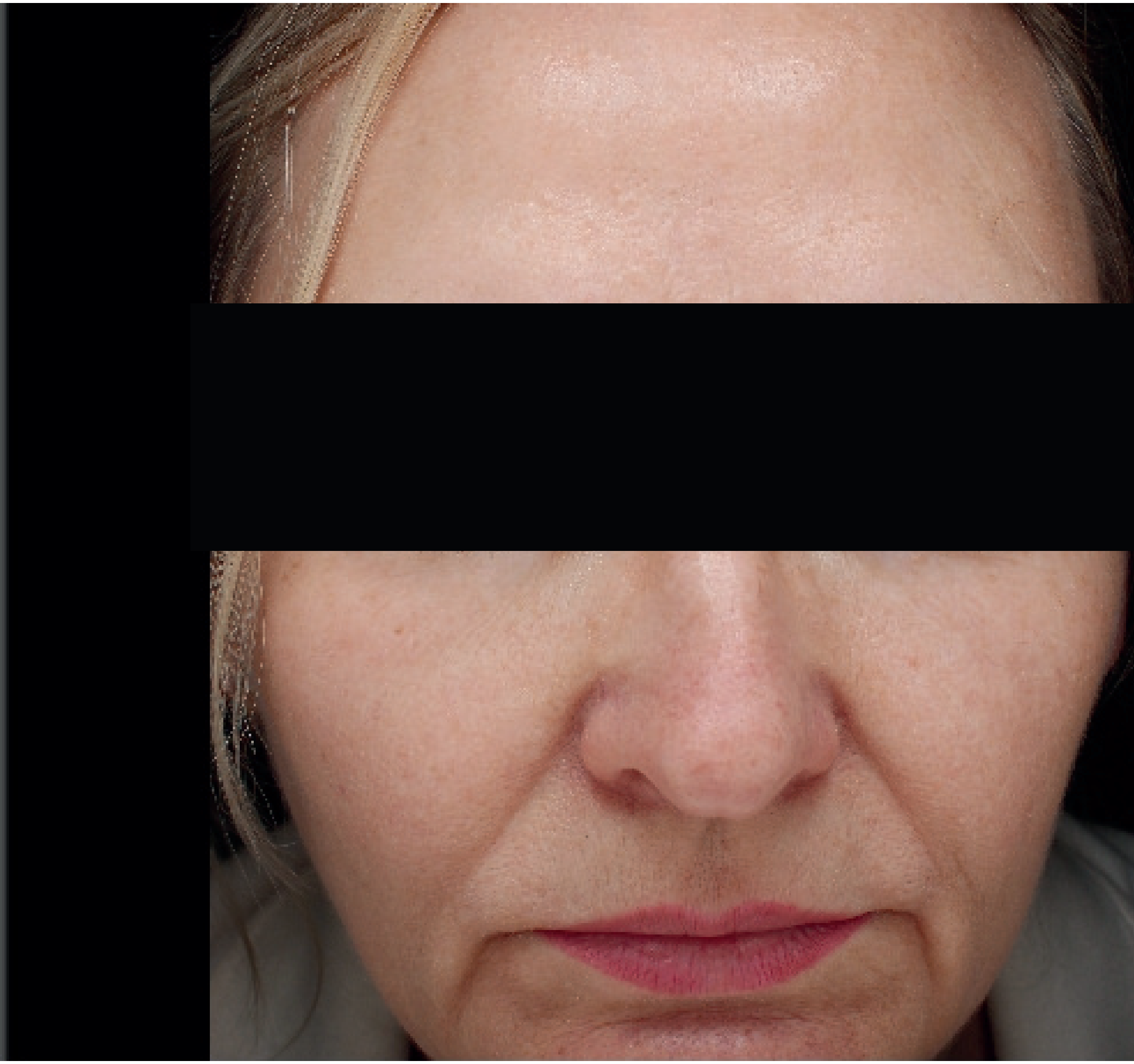

AFTER TREATMENT

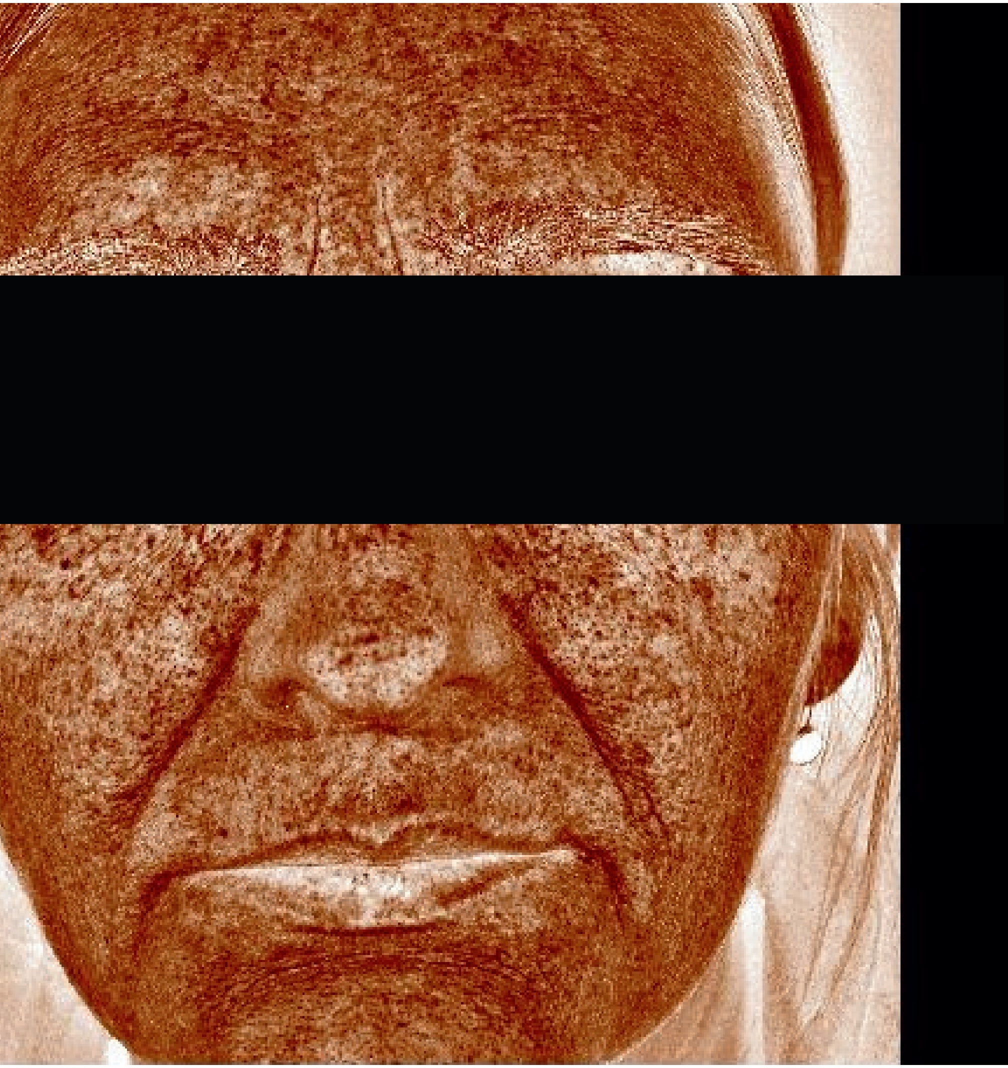

BEFORE TREATMENT

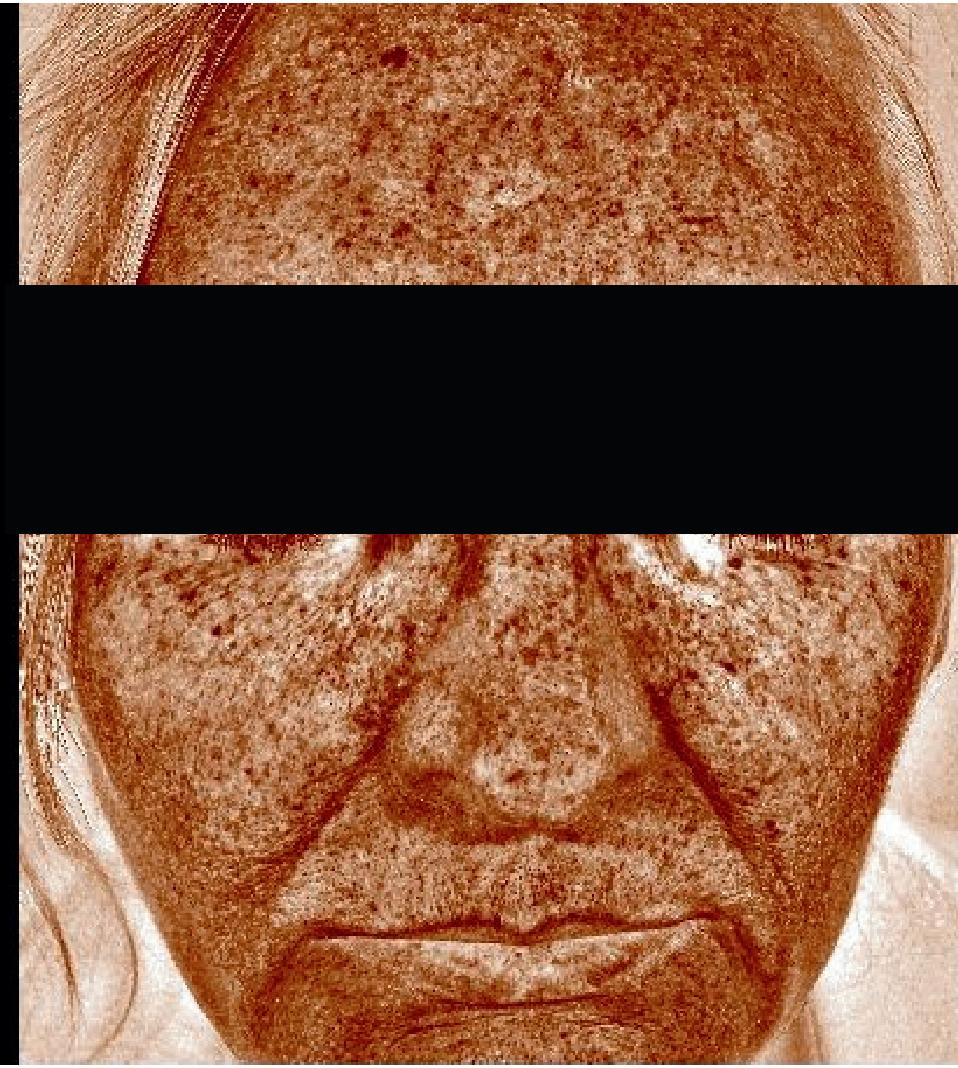

AFTER TREATMENT

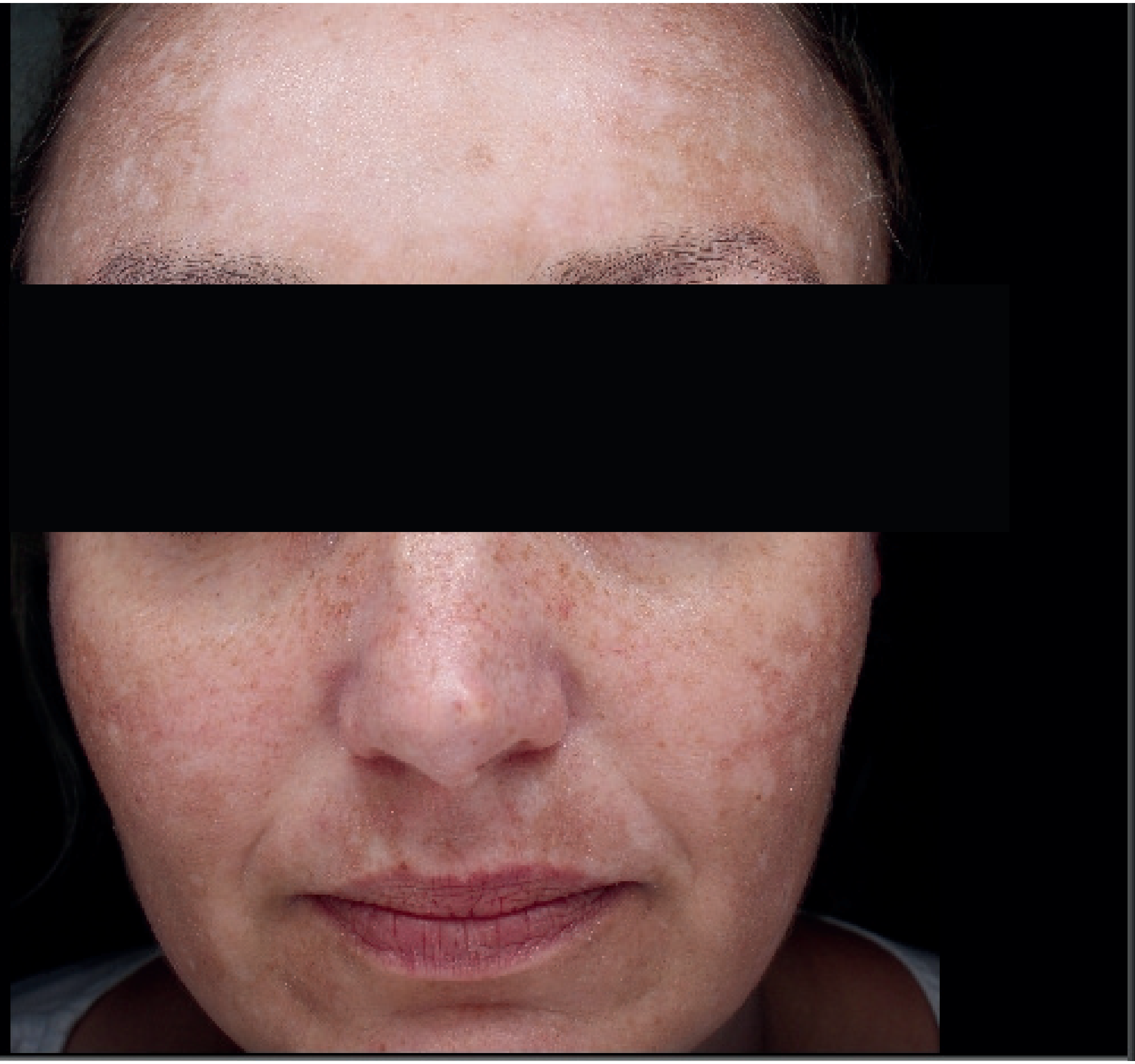

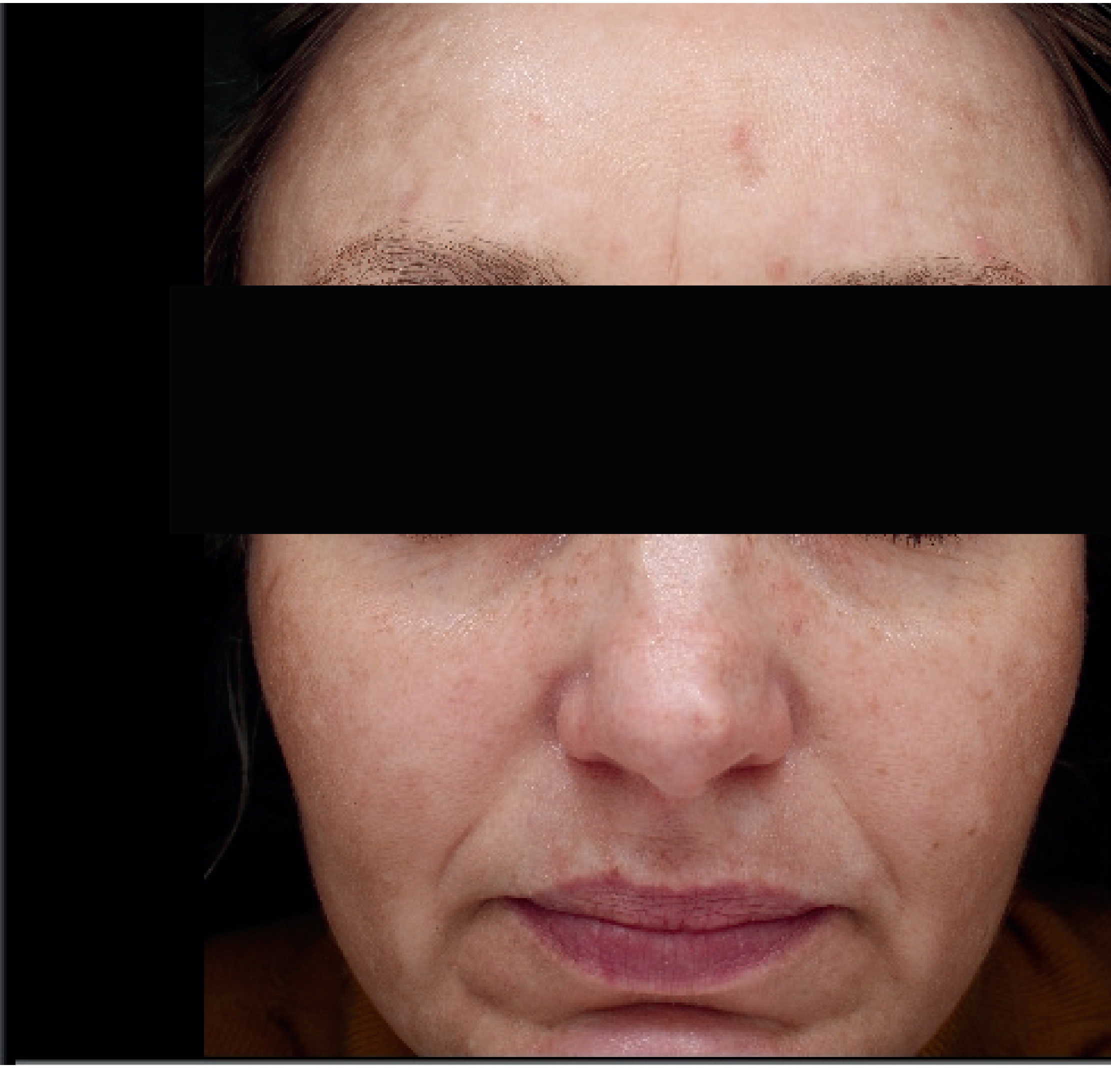

BEFORE TREATMENT

AFTER TREATMENT

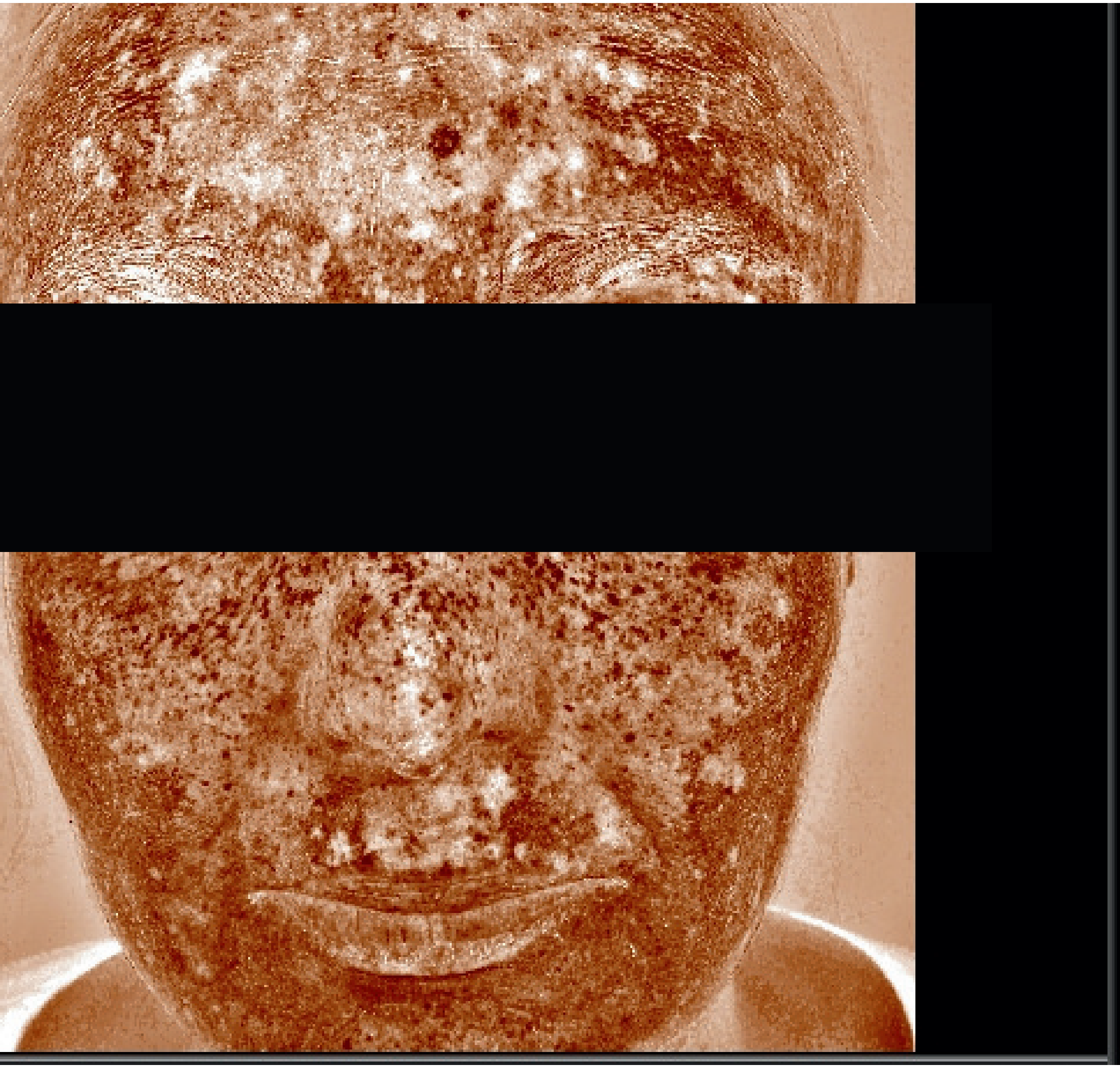

BEFORE TREATMENT

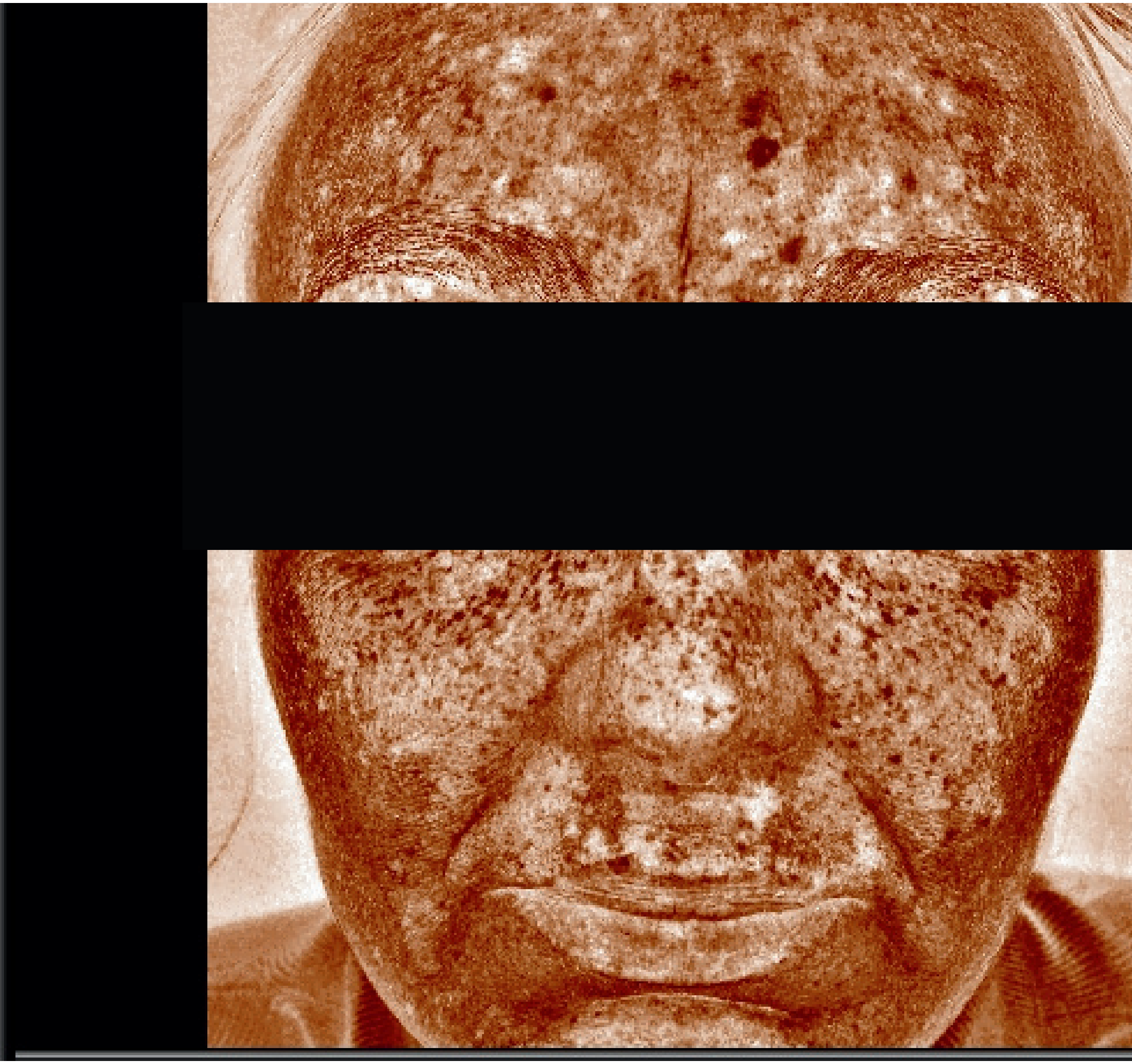

AFTER TREATMENT

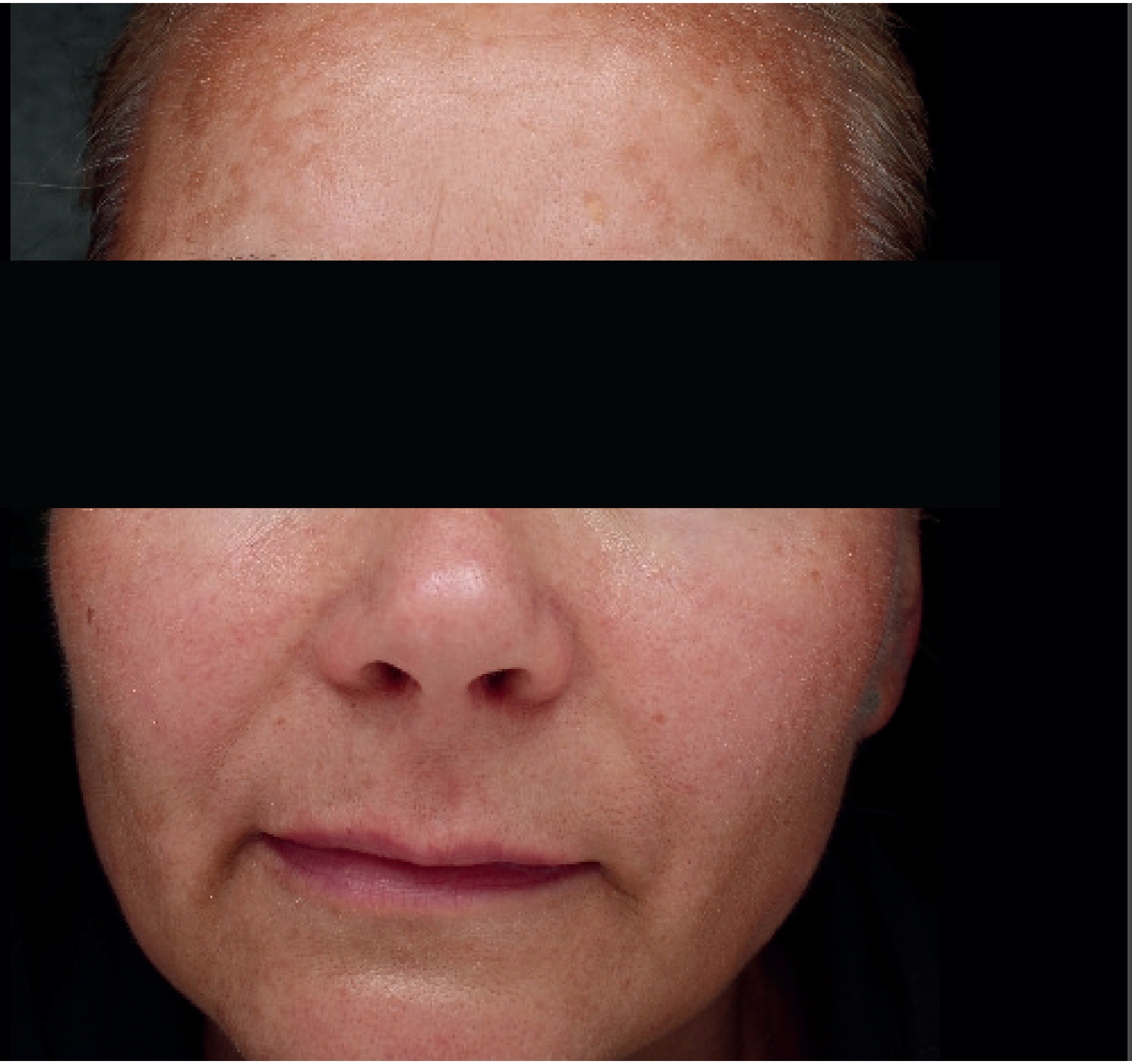

BEFORE TREATMENT

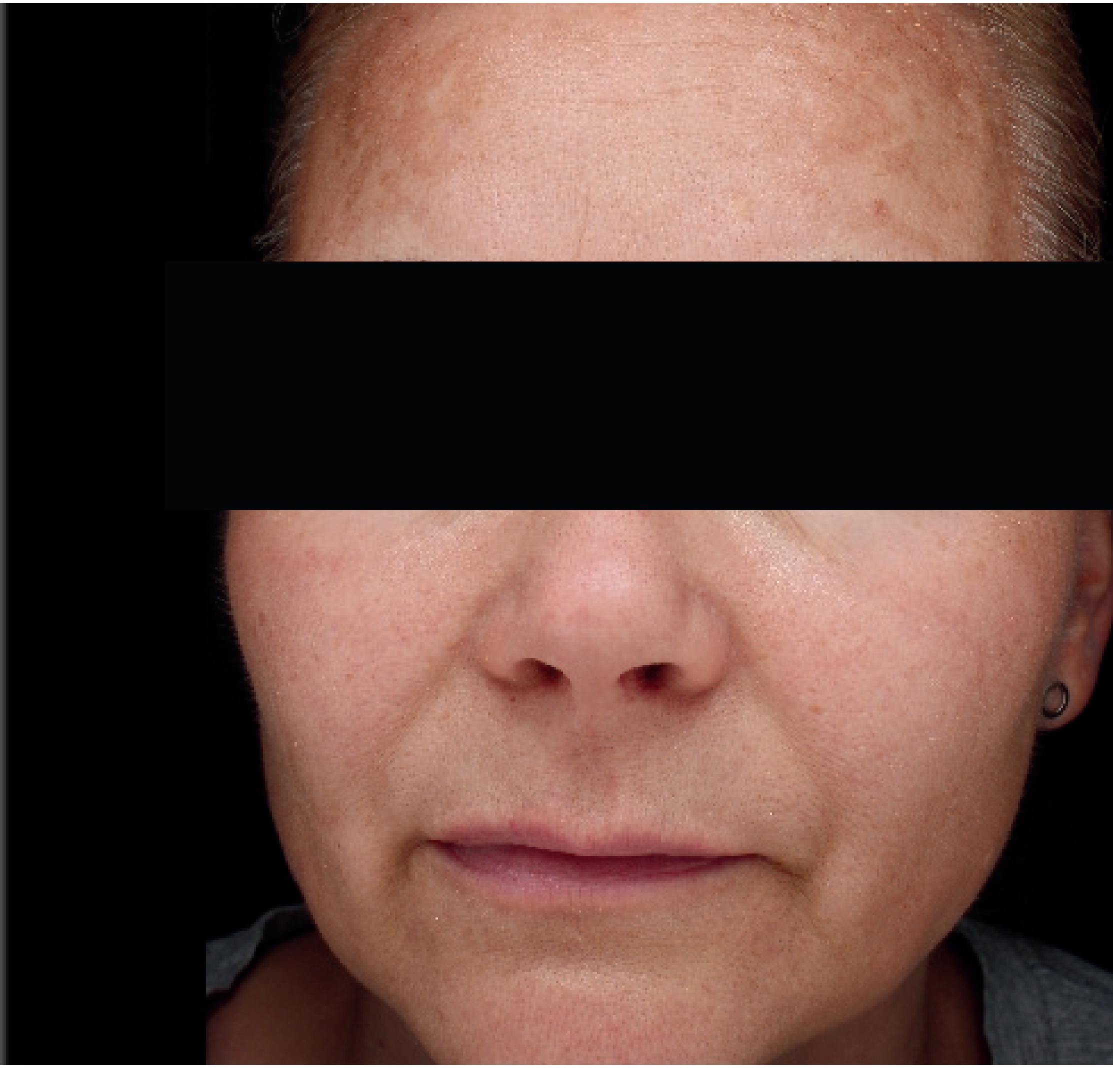

AFTER TREATMENT

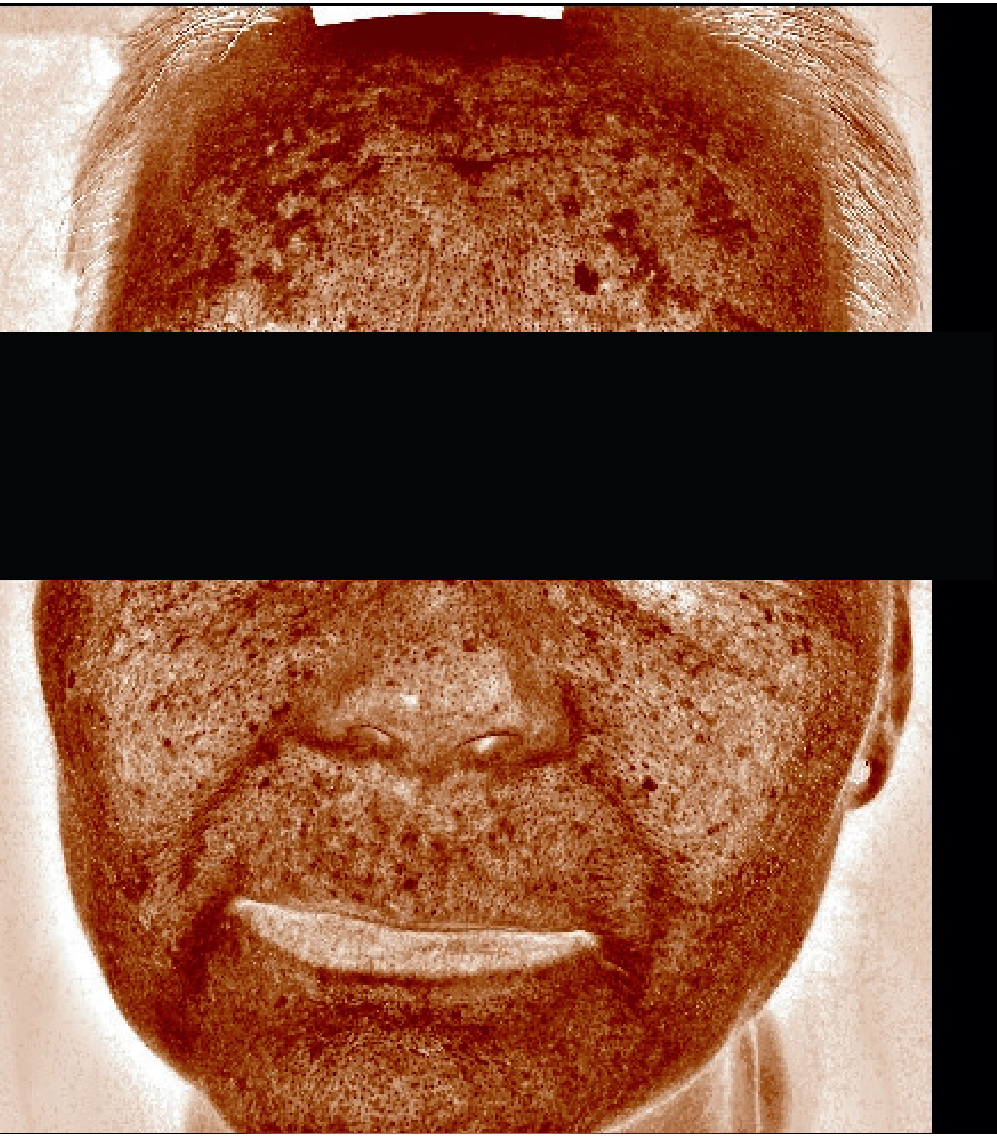

BEFORE TREATMENT

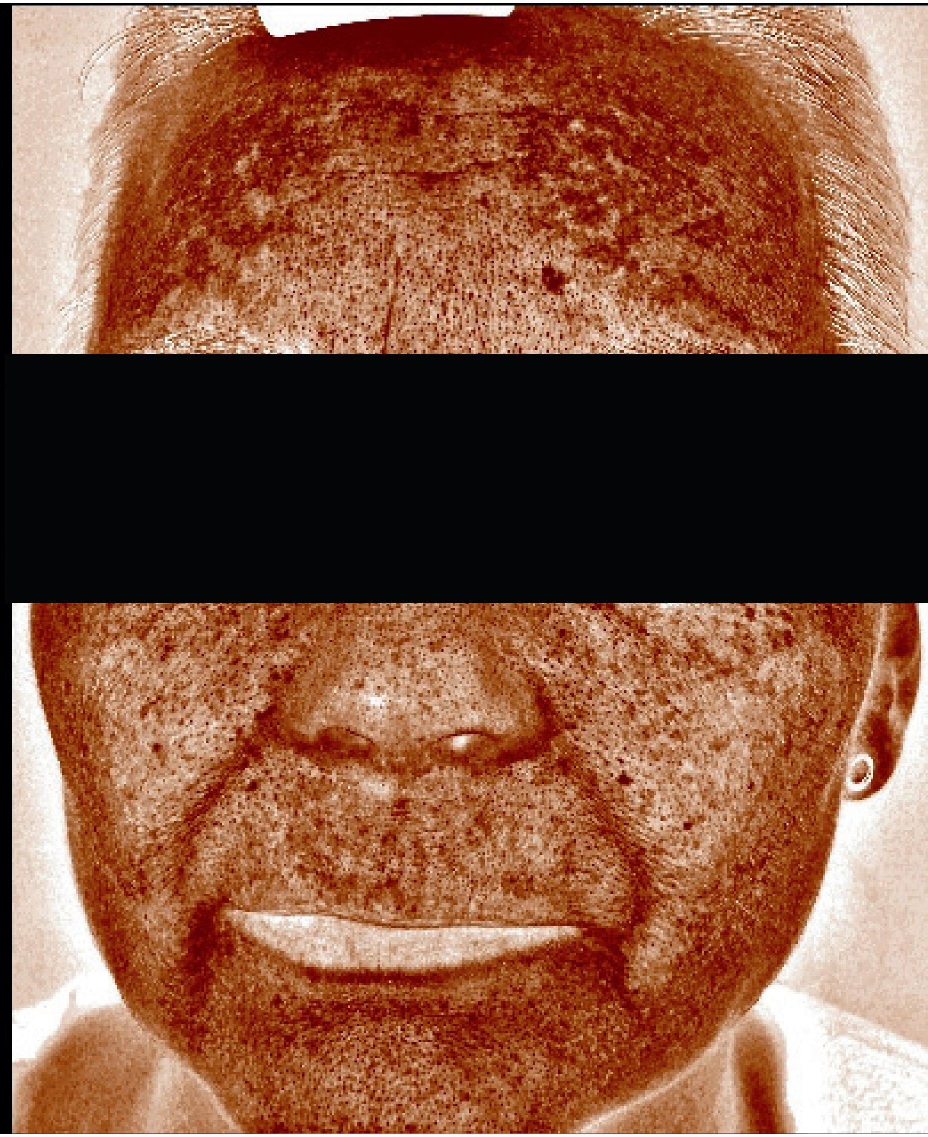

AFTER TREATMENT

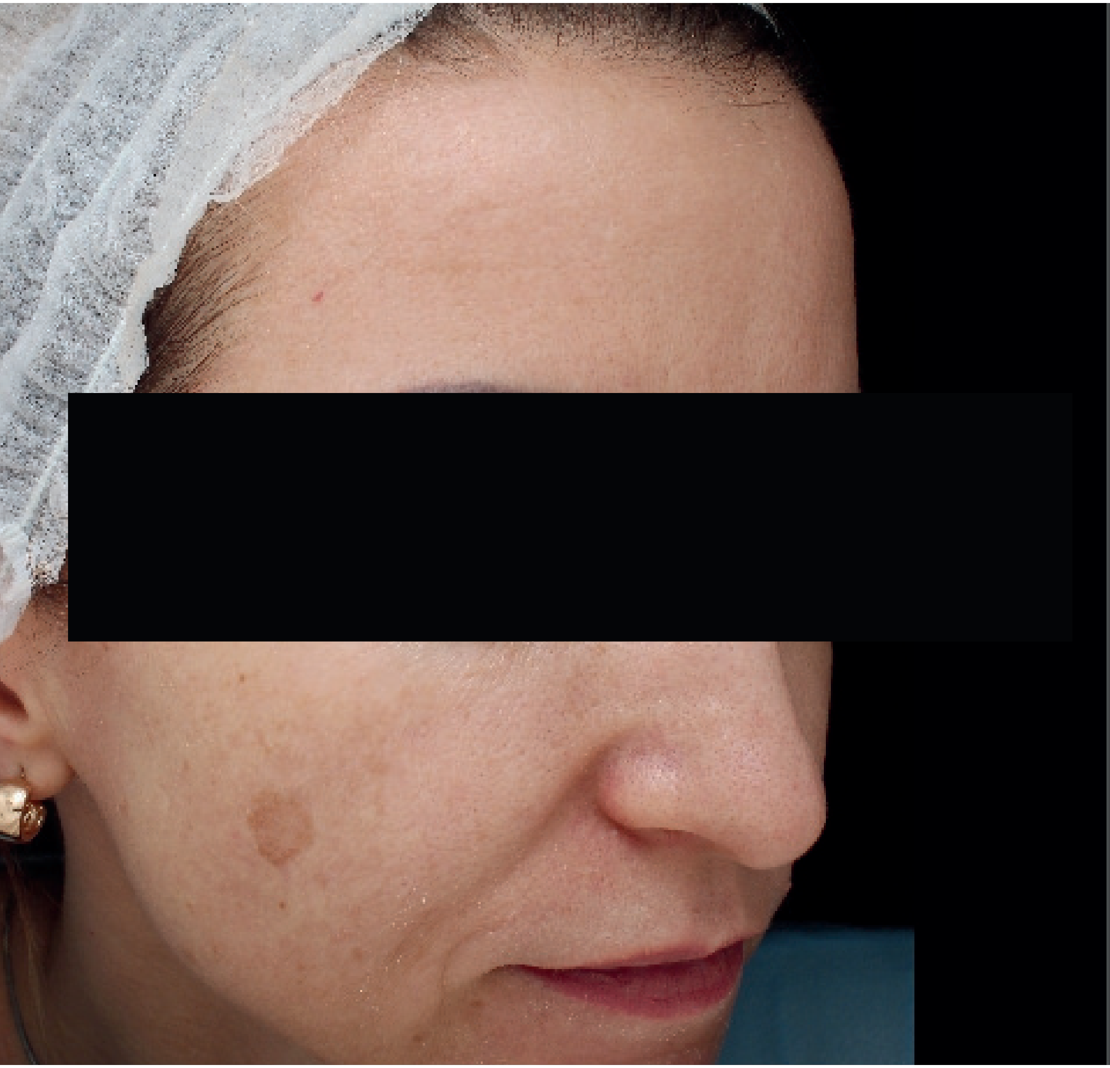

BEFORE TREATMENT

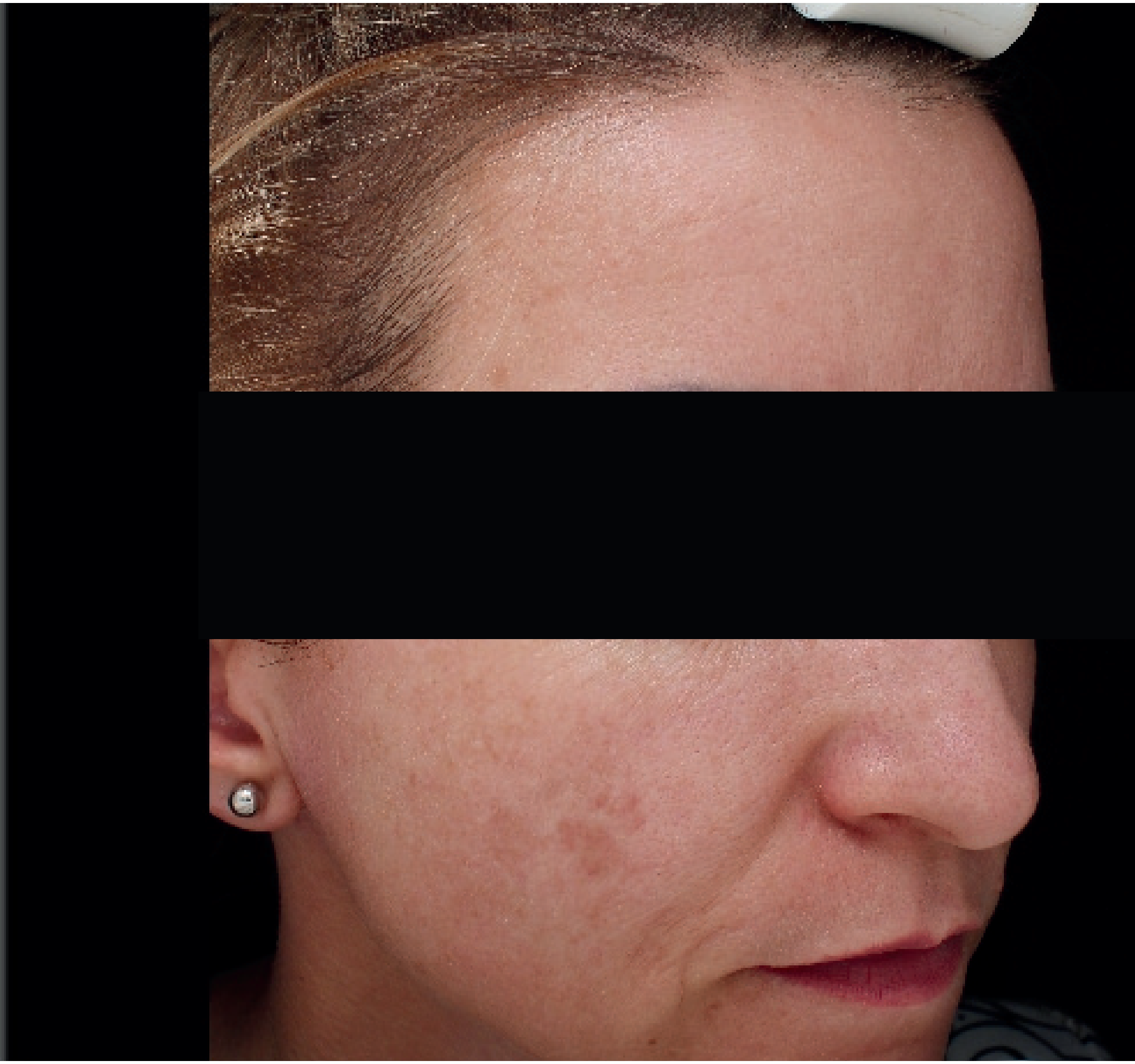

AFTER TREATMENT

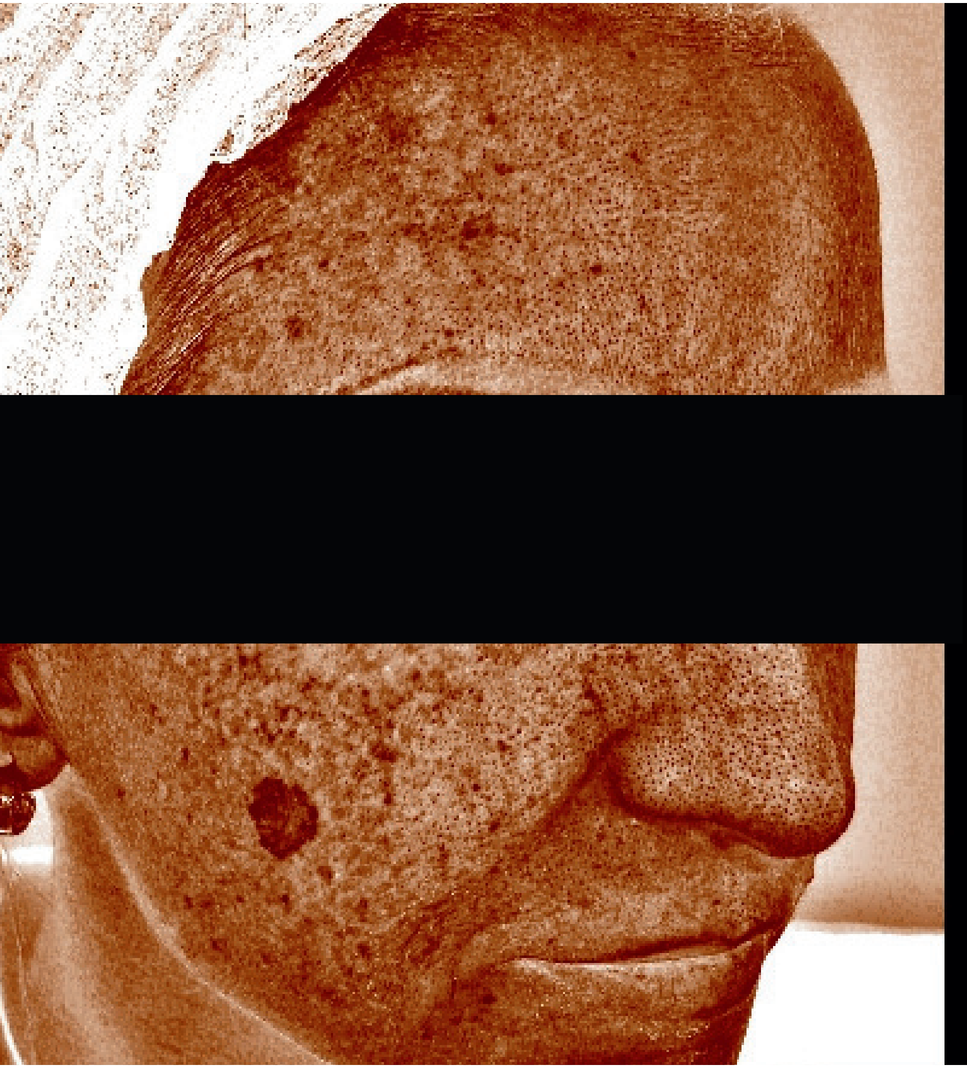

BEFORE TREATMENT

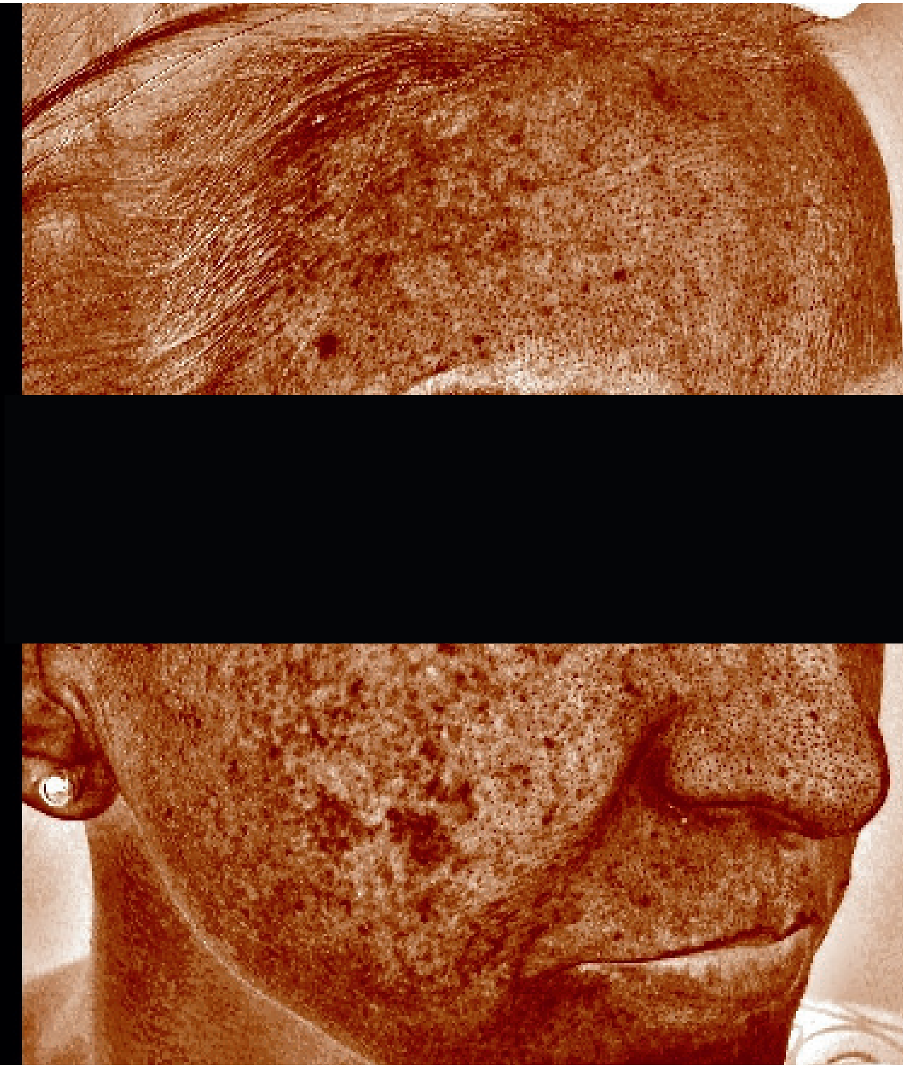

AFTER TREATMENT

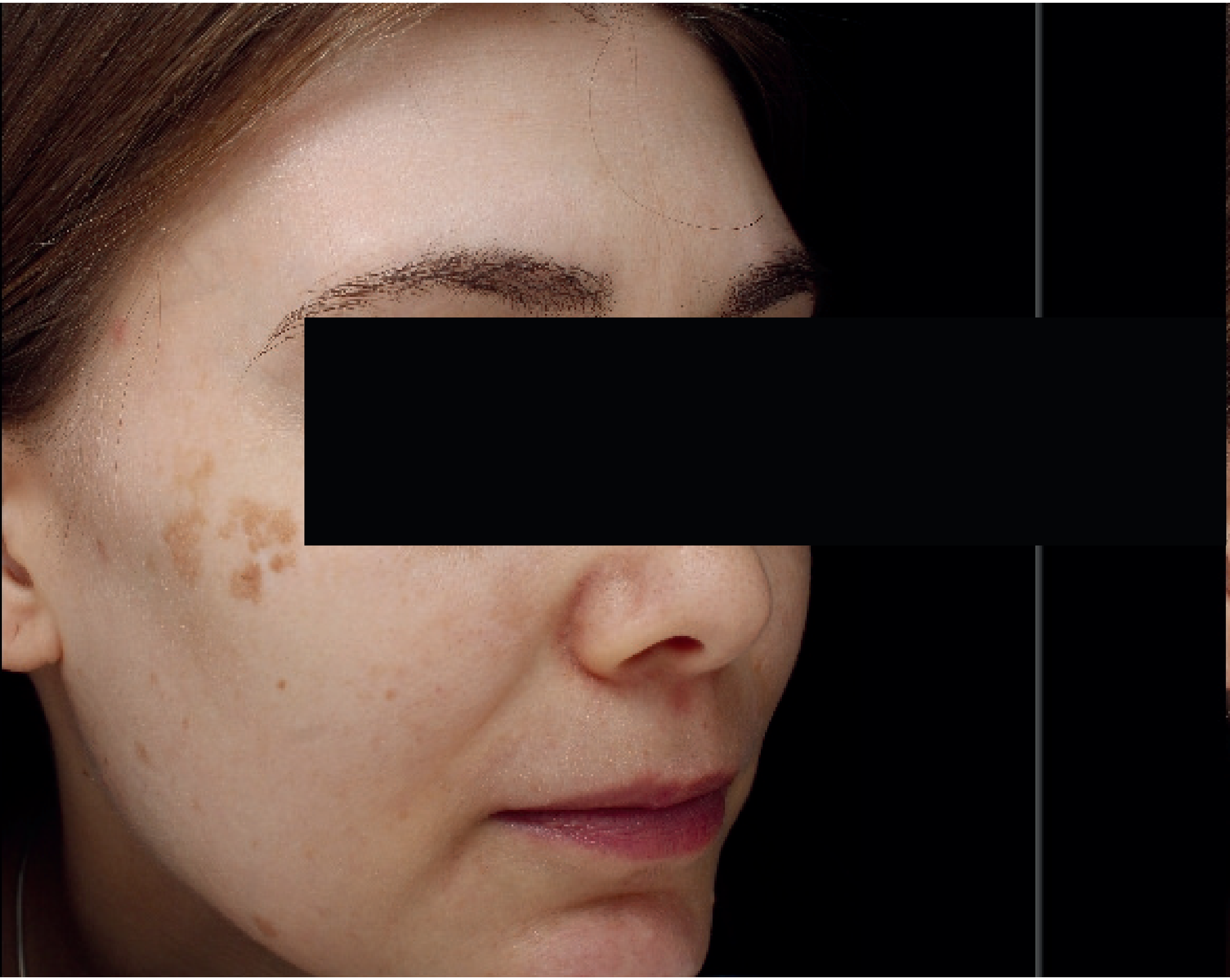

BEFORE TREATMENT

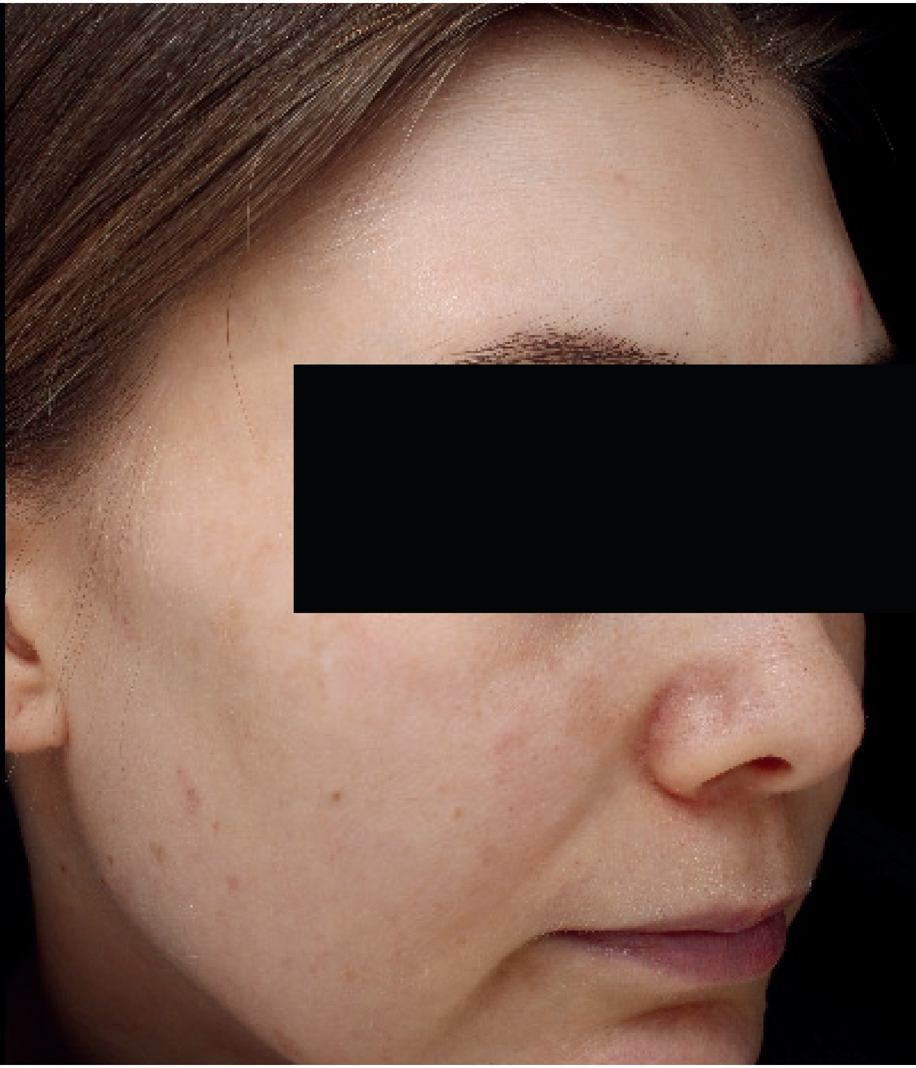

AFTER TREATMENT

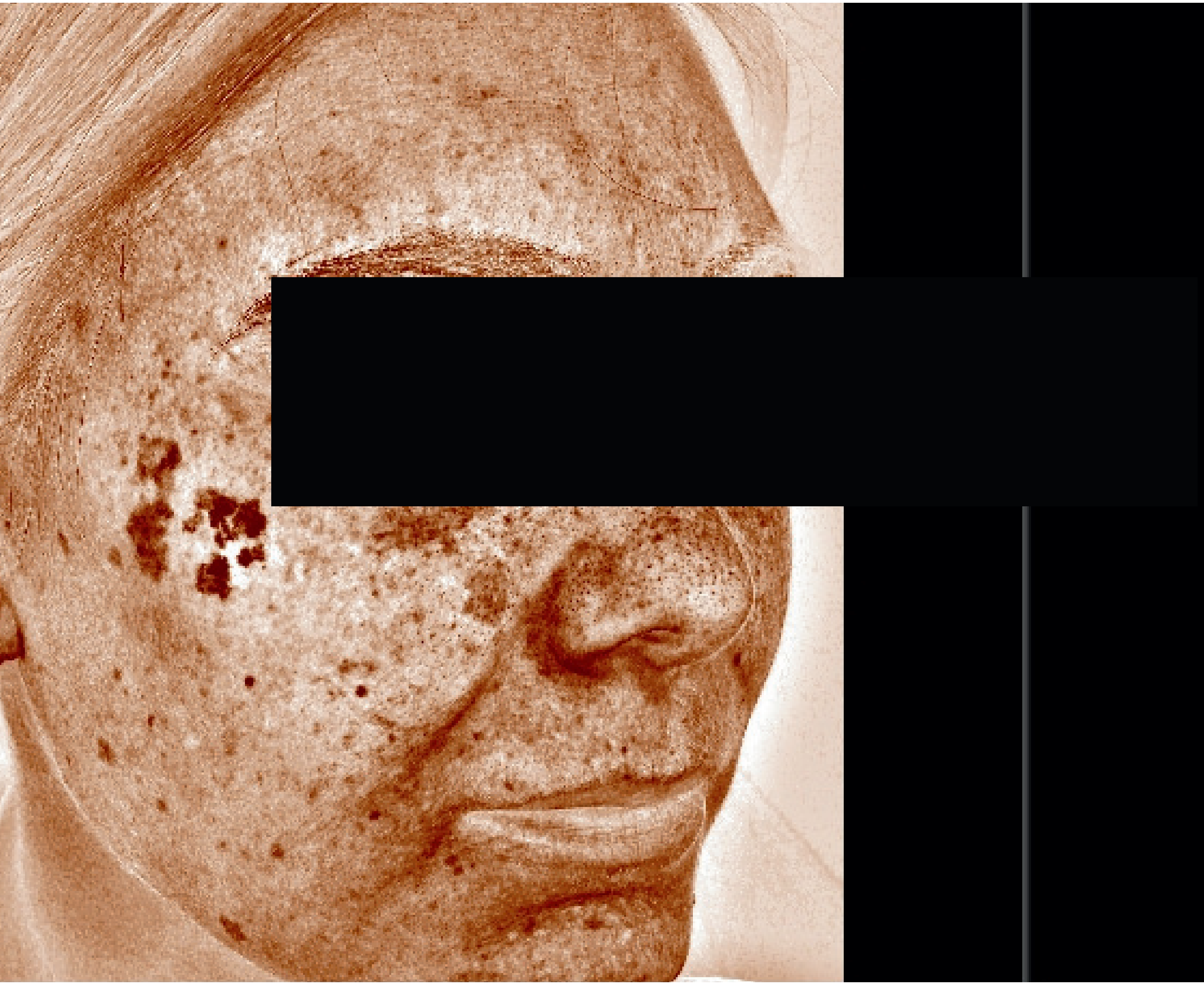

BEFORE TREATMENT

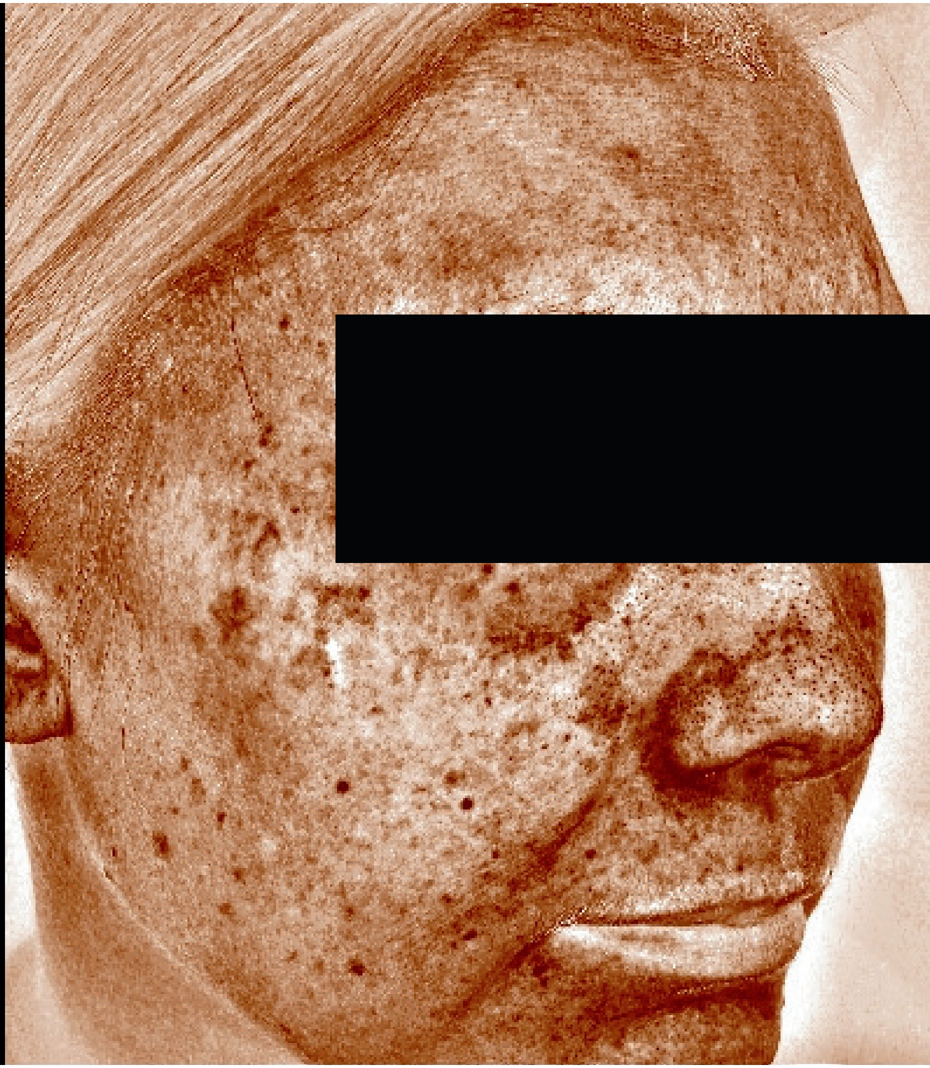

AFTER TREATMENT
